# Dyneins Across Eukaryotes: A Comparative Genomic Analysis

**DOI:** 10.1111/j.1600-0854.2007.00646.x

**Published:** 2007-09-26

**Authors:** Bill Wickstead, Keith Gull

**Affiliations:** Sir William Dunn School of Pathology, University of Oxford South Parks Road, Oxford, OX1 3RE, UK

**Keywords:** Bayesian phylogenetics, dynein, eukaryotic evolution, IAD, IFT, OAD

## Abstract

Dyneins are large minus-end-directed microtubule motors. Each dynein contains at least one dynein heavy chain (DHC) and a variable number of intermediate chains (IC), light intermediate chains (LIC) and light chains (LC). Here, we used genome sequence data from 24 diverse eukaryotes to assess the distribution of DHCs, ICs, LICs and LCs across Eukaryota. Phylogenetic inference identified nine DHC families (two cytoplasmic and seven axonemal) and six IC families (one cytoplasmic). We confirm that dyneins have been lost from higher plants and show that this is most likely because of a single loss of cytoplasmic dynein 1 from the ancestor of Rhodophyta and Viridiplantae, followed by lineage-specific losses of other families. Independent losses in Entamoeba mean that at least three extant eukaryotic lineages are entirely devoid of dyneins. Cytoplasmic dynein 2 is associated with intraflagellar transport (IFT), but in two chromalveolate organisms, we find an IFT footprint without the retrograde motor. The distribution of one family of outer-arm dyneins accounts for 2-headed or 3-headed outer-arm ultrastructures observed in different organisms. One diatom species builds motile axonemes without any inner-arm dyneins (IAD), and the unexpected conservation of IAD I1 in non-flagellate algae and LC8 (DYNLL1/2) in all lineages reveals a surprising fluidity to dynein function.

Dyneins are force-generating adenosine triphosphatases (ATPases) that move along eukaryotic microtubules. One of the key cellular roles of the dynein family is in the movement of the axoneme – the highly conserved microtubule-based structure that provides the motility in all eukaryotic flagella and cilia. Most dyneins so far identified appear to belong to this axonemal class, but there is also a highly conserved cytoplasmic class of dyneins that act as important minus-end-directed motors in processes including vesicle transport, organellar positioning, mitotic spindle organization and chromosome segregation (reviewed in 1–3).

Dynein motors are not homologous to the other eukaryotic microtubule motor superfamily, the kinesins – or to the myosin motors of the actin cytoskeleton – being instead part of the broad ATPase associated with various cellular activities (AAA^+^) superfamily. Dyneins also have both a very different structure and mode of action to that of kinesins. Whereas, the kinesin motor domain is relatively small (∼400 aa) and globular; the dynein motor region is very large (∼3000 aa) and forms a ring of six AAA^+^ modules, the first of which is the site of ATPase activity (4, 5). Structural studies of purified dynein molecules suggest that the dynein motor acts as a winch by rotation of the dynein head, relative to the N-terminal tail (6, 7).

Dynein complexes consist of 1, 2 or 3 dynein heavy chains (DHCs, alternatively often abbreviated as DYH or DYN), which are large polypeptides (∼500 kDa), each containing a single motor domain. The tail region of DHCs binds a variable number of smaller subunits – light chains (LCs; 8–30 kDa), light intermediate chains (LICs; 30–60 kDa) and intermediate chains (ICs; 60–140 kDa) – which regulate dynein complex activity and aid in tethering of cargo. The motor domain of all DHCs is well conserved, but particular kinds of DHC appear to be associated with specific cellular functions. The ICs are also a family of homologous proteins (albeit, slightly less conserved in sequence than the DHC motor) and again have at least some specificity for particular dyneins. Contrastingly, dynein LCs are not a single family of proteins but several. The LCs are also apparently more promiscuous than either ICs or DHCs, with particular LCs being found in several dynein complexes (8).

Here, we have used publicly available genome sequence data from 24 diverse eukaryotes to perform the first assessment of the distribution of DHCs, ICs, LICs and LCs across five of the six proposed eukaryotic supergroups (9). We present a Bayesian phylogeny for the complete repertoire of DHCs and ICs in these 24 organisms, with extensive support from other methods, and compare the distribution of HC families to those of LICs, LCs and IFT proteins.

## Results and Discussion

### Bayesian DHC, IC and LC phylogenies

To assess the dynein repertoire across a broad range of eukaryotes, we selected 24 disparate organisms for which complete or near-complete genome sequences are publicly available. These organisms were: the Metazoa *Homo sapiens*(10), *Takifugu (Fugu) rubripes*(11), *Drosophila melanogaster*(12) and *Caenorhabditis elegans*(13); the yeasts *Saccharomyces cerevisiae*(14) and *Schizosaccharomyces pombe*(15); the Amoebozoa *Entamoeba histolytica*(16) and *Dictyostelium discoideum*(17); the kinetoplastids *Trypanosoma brucei*(18) and *Leishmania major*(19); the diplomonad *Giardia lamblia* (www.mbl.edu/Giardia); the ciliate *Tetrahymena thermophila*(20); the diatoms *Thalassiosira pseudonana*(21) and *Phaeodactylum tricornutum* (www.jgi.doe.gov); the oomycete *Phytophthora sojae*(22); the Apicomplexa *Plasmodium falciparum*(23), *Cryptosporidium parvum*(24) and *Toxoplasma gondii* (www.toxodb.org); the red alga *Cyanidioschyzon merolae*(25); the green algae *Chlamydomonas reinhardtii* (www.jgi.doe.gov) and *Ostreococcus lucimarinus* (www.jgi.doe.gov); and the higher plants *Arabidopsis thaliana*(26), *Populus trichocarpa*(27) and *Oryza sativa*(28). At the time of analysis, a published draft genome sequence was available for all but five of these organisms (*C. reinhardtii*, *G. lamblia*, *P. tricornutum*, *O. lucimarinus* and *T. gondii*). A list of the sources and versions of the data used is given in File S1.

**Figure 1:**
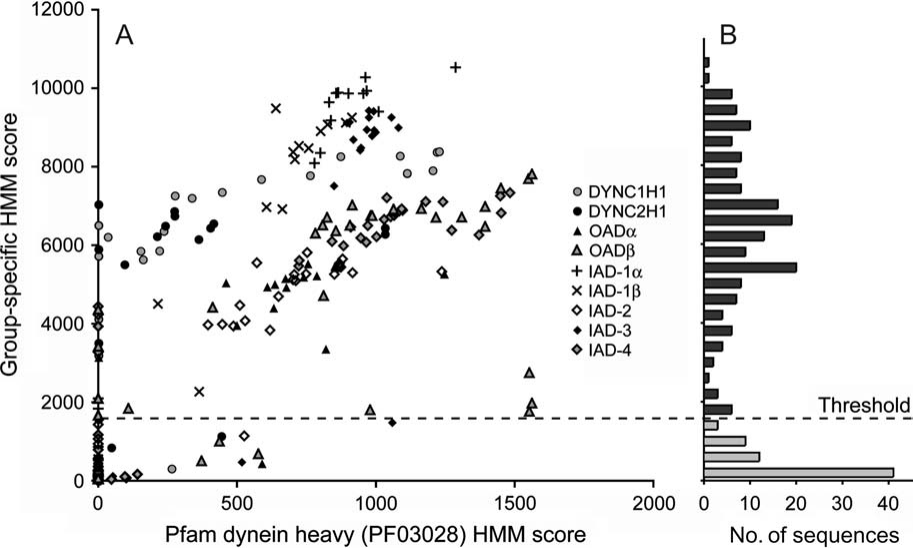
Use of HMMs to identify DHCs encoded in the genomes of 24 diverse eukaryotes. A) Performance of the Pfam dynein heavy HMM, PF03028.5, against group-specific HMMs. The *y*-axis shows score for group-specific HMM giving highest-scoring match. All predicted polypeptides in the 24 genomes with a score >0 on either axis are shown. B) Histogram of the distribution of matches to group-specific HMMs used to define the dynein heavy dataset (score > threshold). On the basis of the distribution, a liberal threshold was chosen to encompass the vast majority of dynein-like sequences without the inclusion of excessive numbers of false positives and extremely divergent sequences that accumulate in the low-score tail.

Using the Pfam ‘dynein heavy’ domain (PF03028) model, we extracted a set of DHCs predicted to be encoded in each of the 24 genomes. Considering the size of the dynein motor domain (the most highly conserved portion of the primary sequences) is ∼3000 aa, the PF03028 model is quite short (783 aa) as it covers only the C-terminal part of the motor domain. Importantly, a number of good candidates for dynein motors do not produce a hit to this model. To ensure that we had the full repertoire of DHCs for each organism, we used this initial set to define new hidden Markov models (HMMs) for specific sets of DHCs and used these to reinterrogate the sequences encoded by the 24 genomes. Figure 1 shows the results of this analysis. As expected, our new, longer models produce higher scores for all sequences (whether DHCs or not). More importantly, the Pfam model PF03028 performs poorly in the identification of particular groups of DHCs – for instance, cytoplasmic dynein 1 and 2 HCs are especially poorly predicted by PF03028 – but produce strong signals with the group-specific models. A number of protistan sequences of all DHC types are also not found by the Pfam dynein heavy model. Both these observations are the result of bias in the databases used in the construction of the Pfam model: bias towards axonemal DHCs (as there are more families than for cytoplasmic dyneins) and towards metazoan sequences (as there are more sequences available). The situation is likely to improve as more varied species are incorporated into the databases, but currently, the model PF03028 misses several DHC sequences that can be clearly identified with our HMMs.

**Figure 2:**
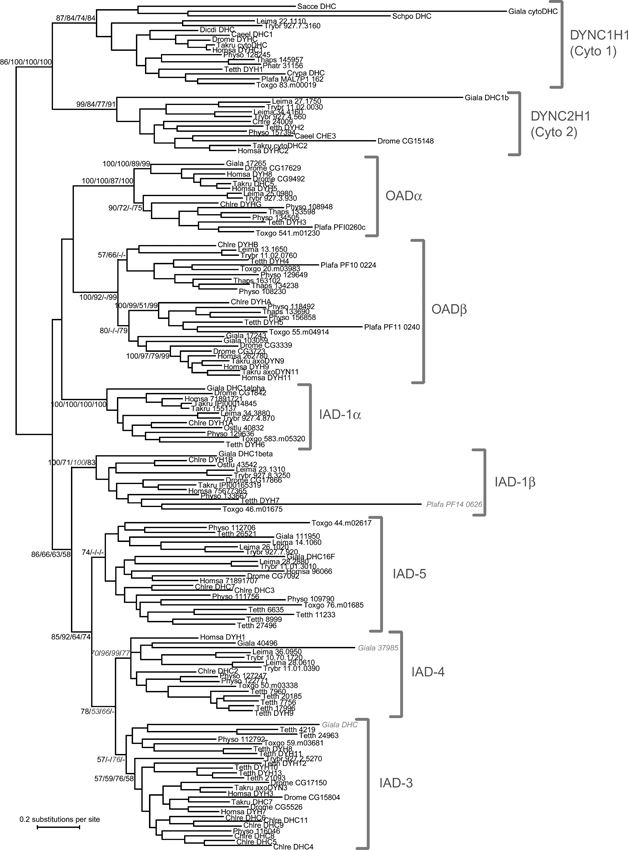
A Bayesian phylogeny for the DHC sequences from 24 diverse eukaryotes. Prefixes: Caeel, *Caenorhabditis elegans*; Chlre, *C. reinhardtii*; Crypa, *C. parvum*; Dicdi, *D. discoideum*; Drome, *D. melanogaster*; Giala, *G. lamblia*; Homsa, *H. sapiens*; Leima, *L. major*; Ostlu, *O. lucimarinus*; Phatr, *P. tricornutum*; Physo, *P. sojae*; Plafa, *P. falciparum*; Sacce, *S. cerevisiae*; Schpo, *S. pombe*; Takru, *T. rubripes*; Tetth, *T. thermophila*; Thaps, *T. pseudonana*; Toxgo, *T. gondii*; Trybr, *T. brucei*. (No DHC: *A. thaliana*, *C. merolae*, *E. histolytica*, *O. sativa* and *P. trichocarpa*). For display, the tree has been rooted by bisecting the longest internal branch,although the true position of the root is unknown. Topology support for selected nodes is indicated (Bayesian partial bootstraps/ML/NJ/MP). Bootstrap values give a conservative estimate of the confidence that a particular group of sequences are monophyletic (94). Generally, groups with >90% bootstrap support were considered to be well supported and those with >70% bootstrap support to have some support. Italicized grey values give clade support, excluding the similarly highlighted sequences. Bootstrap values for all nodes from all four inference methods are given in File S3. Cyto 1, cytoplasmic dynein 1; Cyto 2, cytoplasmic dynein 2.

The DHC homologues can also be identified using BLAST similarity searches, but no small set of sequences can be used to unambiguously detect all DHCs from the complete set of 24 organisms (data not shown), making the group-specific HMMs the best current method to identify DHC sequences. We adopted a liberal score threshold (Figure 1) to extract the complete repertoire of DHCs, predicted to be encoded by the 24 eukaryotes (File S2) and used them to infer a Bayesian phylogeny, supported by partial Bayesian bootstrap replicates and full replicates using maximum-likelihood (ML), neighbour-joining (NJ) and maximum-parsimony (MP) approaches (see *Materials and Methods*). The full DHC phylogeny can be seen in Figure 2, and topology support for all nodes using all methods is supplied in File S3. Our phylogeny is consistent with the classification of DHCs into two groups: cytoplasmic and axonemal. The cytoplasmic group encompasses two families – DYNC1H1 (cytoplasmic dynein 1) and DYNC2H1 (cytoplasmic dynein 2) – of which the latter has the more restricted distribution, being found only in organisms that build axonemes at some point in their life cycle (see below). Previous works have divided the axonemal dyneins into six families – outer-arm dynein (OAD)α, OADβ, OADγ, inner-arm dynein (IAD)-1α, IAD-1β and the single-headed dyneins (29). More recent work has suggested that there may be more families within the single-headed category (30). The analysis presented here utilizes the data emerging from several recent eukaryotic genome sequencing projects along with sophisticated phylogenetic methods to greatly extend these analyses. This extended analysis suggests that the OADα, OADβ and OADγ groups in fact only encompass two well-supported ancestral families, members of both of which are found in all organisms included in our analysis that build motile axonemes (discussed in more detail below). Our analysis also suggests that the single-headed DHCs can be classified into three distinct families. Here, we call these groups IAD-3, IAD-4 and IAD-5 to follow on from the two 2-headed IAD-1 families. The distribution of these dynein families is discussed in more detail below.

The core of most of the DHC groups is well supported by all methods used (see File S3); although, the placing of three divergent sequences (Plafa_PF14_0626, Giala_37985 and Giala_DHC; indicated in Figure 2) is not consistent between methods and shown be treated with caution. However, all of the DHC sequences can be placed into one of the nine dynein families identified here with reasonable confidence.

**Figure 3:**
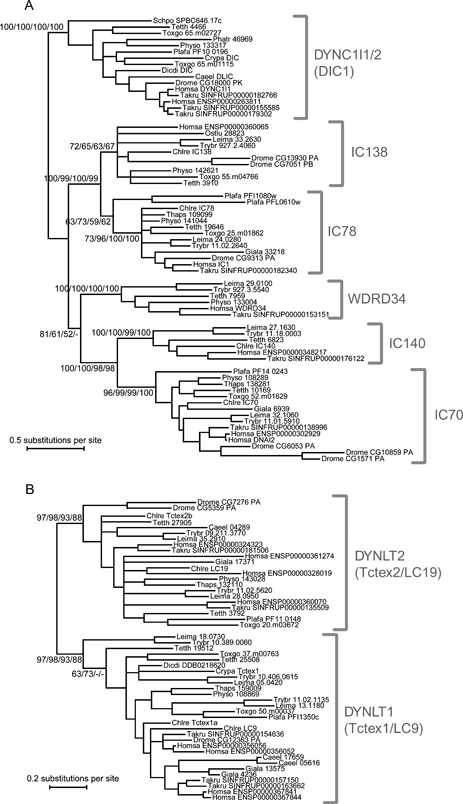
Bayesian phylogenies for: A) dynein IC and B) Tctex1/Tctex2 family LC sequences from 24 diverse eukaryotes. Prefixes as in legend to Figure 2. For display, trees have been rooted by bisecting the longest internal branch. Topology support for selected nodes is indicated (Bayesian partial bootstraps/ML/NJ/MP). Bootstrap values for all nodes under all four methods are given in Files S4 and S5.

To complement the DHC analysis, we also inferred phylogenies for the dynein IC superfamily and the multigene Tctex1/Tctex2/LC9/LC19 (DYNLT1/2) LC family (Figure 3 and Files S4 and S5). From the same dataset of 24 eukaryotes, we used an iterative HMM searching approach (see *Materials and Methods*) to identify 67 non-redundant ICs and 51 non-redundant DYNLT1/2 LCs from the predicted proteomes of these organisms and used them to infer Bayesian phylogenies (again supported by ML and NJ approaches). On the basis of these phylogenies, ICs can be divided into six well-supported groups – DYNC1I1/2 [dynein intermediate chain1 family (DIC1)], IC70 (IC2/ODA6), IC78 (IC1/ODA9), IC140 (IDA7), IC138 (BOP5) and a new IC family WDRD34 (named for the human WD-repeat domain 34-containing protein it contains). As for DHCs, there is an apparent division between cytoplasmic and axonemal clades. The DYNC1I1/2 family contains sequences known to be components of the cytoplasmic dynein 1 (DYNC1) complex. In contrast, it has been shown that in *Chlamydomonas*, IC70 and IC78 are components of the OAD complex (31–35), whereas IC138 and IC140 associate with IAD I1 (36–38). The newly identified clade WDRD34 contains sequences that are, as far as the authors are aware, of unknown function.

The Tctex1/Tctex2/LC9/LC19 phylogeny encompasses just two well-supported clades – Tctex1/LC9 (DYNLT1) and Tctex2/LC19 (DYNLT2). Several organisms encode more than one example of each family, but generally in the analysis presented here, they do not fall into well-supported subgroups in contrast to the situation suggested by previous more limited phylogenies (39). Only the Tctex1/LC9 (DYNLT1) family contains non-flagellate organisms on the basis of this analysis.

### No dyneins in higher plants, red algae or Entamoeba

Lawrence et al.(40) have previously noted that there are no DHCs in the genome sequence available for *Arabidopsis*, leading to the suggestion that higher plants have dispensed with the dynein motor. This was subsequently called into question by the identification of four non-redundant sequences from DHC genes (encoding axonemal dynein family members) in the shotgun sequence generated as part of the rice (*O. sativa*) genome project, (41) raising the possibility that the situation may be more complicated than the *Arabidopsis* sequence analysis might imply. However, as the sequencing of the rice genome has progressed, these sequences have failed to assemble into any of the large contigs – the version 5 assembly, released January 2007 (http://www.tigr.org/tdb/e2k1/osa1/), covers 372 Mb of the estimated 430 Mb euchromatic genome – and no additional reads with good similarity to DHCs have emerged (data not shown). This makes it highly likely that the potential DHC gene sequences found during the rice genome sequencing effort are from contaminating DNA and not the plant itself.

**Figure 4:**
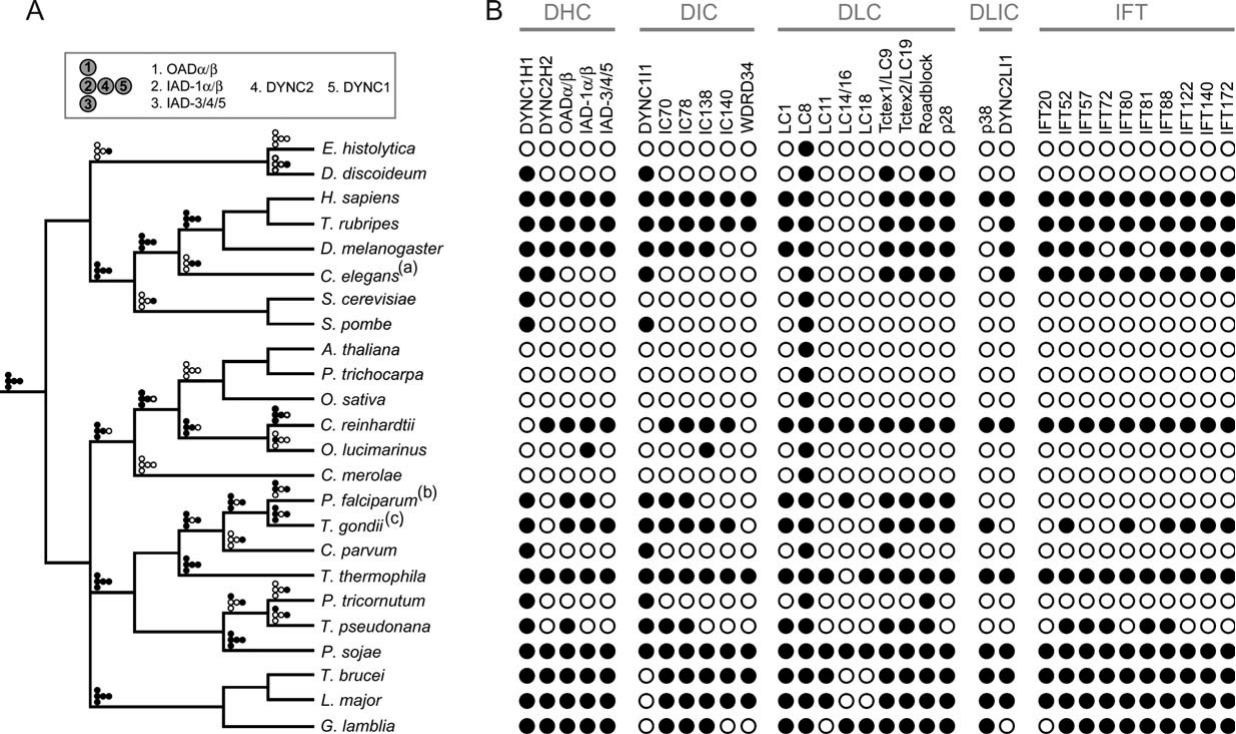
The distribution of dynein and IFT components across 24 diverse eukaryotes. A) Cladogram showing the likely evolutionary relationships of the organisms analysed and the inferred DHC repertoire in ancestral organisms (changes to the repertoire are shown). B) Presence (dot) or absence (circle) of identifiable orthologues of five DHC classes (DHC); six IC groups (DIC); nine LC groups (DLC); one LIC (DLIC); and 10 components of the IFT system (IFT). The names LC11, LC14, LC16, LC18 and LC19 refer to the LCs identified as ‘*M*_r_= 11 000’, ‘*M*_r_ = 14 000’, etc., isolated from *Chlamydomonas* flagella (47, 74, 95). Orthologues were identified by RBB analysis (File S7) and, where necessary, iterative-HMM searches followed by phylogenetic inference (Figure 3). Grey dots indicate sequences failing the iterative-HMM cutoff but giving reciprocating BLAST-hits. Organisms building flagella/cilia are shown in bold. Notes: (a) *Caenorhabditis elegans* cilia are immotile; (b) *Plasmodium falciparum* builds flagella by an IFT-independent mechanism; (c) Briggs et al. (56) suggest that there may be cryptic orthologues of IFT57 and IFT72 encoded in the *T. gondii* genome that are not found in the predicted protein datasets used here.

We find no evidence in the assembled *Arabidopsis*, *Populus* or rice genomes for genes encoding DHCs. This is true, both for the predicted protein dataset and when using tBLASTn searches of the DNA itself (data not shown). Moreover, we find no good evidence for ICs, LICs or any LCs, except LC8 (DYNLL1/2). The presence of LC8 has been previously noted in plants, and this LC is notable in being highly conserved in all 24 eukaryotic genomes analyzed here, regardless of DHC complement (Figure 4B). Taken together, these data strongly suggest that the original observation of the lack of dyneins in higher plants (40) is correct.

Not only have flowering plants dispensed with the dynein family of motors but also our analysis suggests that the loss of cytoplasmic dynein 1 (DYNC1) predates the divergence of the Archaeplastida (i.e. the land plants, green and red algae and glaucophytes). This can be seen in the lack of genes encoding either DYNC1H1 or DYNC1I1/2 families in the genomes of the red alga *C. merolae* and green algae *C. reinhardtii* and *O. lucimarinus*– although *Chlamydomonas* has retained cytoplasmic dynein 2 [Figure 2 and (42)]. *Chlamydomonas* has retained the dynein families associated with the flagellum (see below), and alongside the 15 DHCs in the analysis shown in Figure 2– plus one protein (id: 37963) that is 100% identical to Chlre_DYHG – the Joint Genome Institute (JGI) protein models for this organism (v3.0; http://genome.jgi-psf.org/Chlre3/) include one fragmentary sequence that was too short to be included in the analysis. We were concerned that this protein (id: 115120) might be a fragment of a DYNC1H1 protein. While only fragmentary in the assembly v2.0/v3.0 protein models (and the v3.1 gene catalog), there is a ‘JAMBOREE’ annotation for this *Chlamydomonas* locus that encodes a full length DHC (id: 206178). Phylogenetic inference using this sequence and a subset of dynein sequences from the larger DHC phylogeny shows clearly that this sequence does not represent a ‘missing’ DYNC1H1 but is a divergent member of the axonemal dyneins (File S6). It thus appears that *Chlamydomonas*, which is such an important model for axonemal dynein action, lacks the cytoplasmic dynein counterpart.

Although the flagellate *C. reinhardtii* has retained all dynein families except cytoplasmic dynein 1, the non-flagellate red alga *C. merolae*– like *Arabidopsis*, *Populus* and rice (and presumably all other angiosperms) – has no dynein motors at all. In the light of these data, we believe that the isolation by polymerase chain reaction of DHC gene fragments putatively from *Nicotiana*(43) should be treated with caution; there are no identifiable DHC sequences currently in the publicly available *Nicotiana tabacum* expressed sequence tag (EST) sequence database (www.estarray.org).

Interestingly, flowering plants and red algae are not the only lineages to have dispensed with dyneins altogether. As well as the apparent loss of DYNC1 in the ancestor of the Archaeplastida, a second, independent loss of DYNC1 has occurred in *E. histolytica*– leaving this non-flagellate organism also devoid of any dynein motors. In agreement with these data, *E. histolytica*, *C. merolae*, *A. thaliana*, *P. trichocarpa* and *O. sativa* genomes are all free of identifiable homologues of dynein ICs and all LCs except LC8 (Figure 4B). The occurrence of LC8 (DYNLL1/2) in organisms not possessing dynein motors demonstrates that although this LC is a component of DYNC1 (44, 45) and also axonemal dyneins and radial spokes (8, 45–48), its role is not limited to functions associated with dynein. Interestingly, the budding yeast LC8 orthologue, Dyn2p (YDR424C), is found in cytoplasmic dynein complexes but also associated with nuclear pore components (49).

As might be expected, the occurrence of the DIC1, which contains both mammalian DYNC1I1 and DYNC1I2 proteins, follows the presence of DYNC1 HC reasonably well. However, we found no good homologue of DYNC1I1/2 in budding yeast (*S. cerevisiae*) or in the three Excavata (*T. brucei*, *L. major* and *G. lamblia*) – each of which have apparently dispensed with this protein without losing DYNC1 in its entirety (Figure 4B). The *S. cerevisiae* protein Pac11p is a WD-repeat-containing protein (as are dynein ICs) that associates with dynein (50) and has been proposed to be the budding yeast orthologue of DYNC1I1/2 (51). However, Pac11p is less similar to the ICs from other organisms in our analysis than are other WD-repeat-containing proteins from yeast, which are very unlikely to be dynein subunit (File S7). Pac11p also fails to form a reciprocal-best-BLAST (RBB) match to any of the other ICs (File S8). It is thus not clear from our analysis if Pac11p is a very divergent DYNC1I1/2 family member or a different WD-repeat protein co-opted into the dynein complex. We also found no good homologue of the LC Tctex1/LC9 (DYNLT1) family in yeast (Figure 4B). This LC is missing from organisms that lack all DHCs (and also the green alga *Ostreococcus*, which is a special case, as described below). However, its presence in *Chlamydomonas* implies that it is not specific for DYNC1.

### Cytoplasmic dynein 2 and intraflagellar transport

Heavy chains of cytoplasmic dynein 2, known as DHC1b in *Chlamydomonas* and DYNC2H1 in the new mammalian nomenclature (52), are more similar at the level of primary sequence to the DYNC1H1 family than to axonemal HCs (data not shown) and form a single clade with the DYNC1H1 family on an unrooted tree (Figure 1). However, the DYNC2H1 family is monophyletic and distinct from that of DYNC1H1. In spite of its name, the primary role of cytoplasmic dynein 2 is in building and maintaining cilia/flagella as part of the intraflagellar transport (IFT) machinery – providing the essential retrograde motor to complement the anterograde movement of motors of the kinesin-2 family (reviewed in 53). The dominance of this role is evident in the distribution of DYNC2 HCs: absent from all organisms that don’t build cilia/flagella at some stage in their life cycle. However, our phylogeny reveals that dyneins of this type are also absent from some flagellum-building organisms: *P. falciparum*, *T. gondii* and *T. pseudonana*. This finding does not appear to be a result of erroneous gene models or genome sequence gaps because these three organisms also lack the LIC, D1bLIC (DYNC2LI1; Figure 4B), which is specific for DYNC2 (54). Given the most likely evolutionary relationships between the organisms (Figure 4A), the most parsimonious explanation for the observed distribution of DYNC2 would be a single loss in the common ancestor of *P. falciparum* and *T. gondii* and a second independent loss in *T. pseudonana*.

The microgametes of the malaria parasite *P. falciparum* build their flagella in the cytoplasm (55), and Briggs et al. (56) have previously demonstrated that this occurs through an IFT-independent mechanism and that *P. falciparum* lacks IFT proteins (including kinesin-2 motors). Therefore, the finding here of an absence of DYNC2 in *P. falciparum* is predictable based on the known biology of this organism and was expected. However, the lack of DYNC2 in *T. gondii* and *T. pseudonana* was entirely unexpected as both organisms possess at least some of the central components of IFT, including the kinesin-2 motor [Figure 4B and (56, 57)]. As this would strongly suggest that IFT is still active in these organisms, it raises the obvious question as to what in these organisms is providing the retrograde motor function? Alternatively, is there something about the biology of these organisms – which build flagella only as part of gametogenesis – that relieves the need for a retrograde motor?

### Axonemal dyneins

Most dyneins belong to the axonemal class. In contrast to the mere two families of cytoplasmic DHCs, we find seven identifiable well-supported families of axonemal DHCs. Unsurprisingly, these axonemal families are mostly restricted to organisms that build motile flagella or cilia at some stage of their life cycle (see above for discussion of reports of axonemal dyneins in higher plants and below for discussion of *Osteococcus*). There have been multiple losses of axonemes during eukaryotic evolution, and there have been multiple concomitant losses of axonemal dyneins. This is in contrast to the limited number of losses of cytoplasmic dynein 1. However, from the distribution of DHC families in extant eukaryotes (Figure 4), it is likely that the common ancestor of all eukaryotes had a functioning axoneme and possessed all the DHC families described in this work. Specific lineages have then lost, rather than gained, particular families and functions. The nematode *C. elegans* is an interesting case as it builds cilia in certain, differentiated cell types, but they are all immotile. In keeping with this biology, the *C. elegans* genome encodes homologues of both cytoplasmic dynein families, and also a footprint for the IFT system, but no axonemal DHCs.

Axonemal DHCs can be grouped into three classes: HCs of the axonemal OADs, 2-headed IADs and single-headed IADs. The OADs have been previously described as encompassing three DHC families – OADα, OADβ and OADγ(29). Rather confusingly, the nomenclature for the OADs is not consistent between species (for example, *Chlamydomonas* OADα is the orthologue of *Tetrahymena* OADγ, and *Chlamydomonas* OADγ is the equivalent of *Tetrahymena* OADα). Our more extensive phylogeny provides strong support for only two ancestral groups: (i) the OADα family – encompassing the innermost of the OADs from all species – including *Tetrahymena* OADα (Tetth_DYH3), metazoan OADα and *Chlamydomonas* OADγ; and (ii) the OADβ family – encompassing both *Tetrahymena* OADβ (Tetth_DYH4) and OADγ (Tetth_DYH5; orthologous to *Chlamydomonas* OADα).

**Figure 5:**
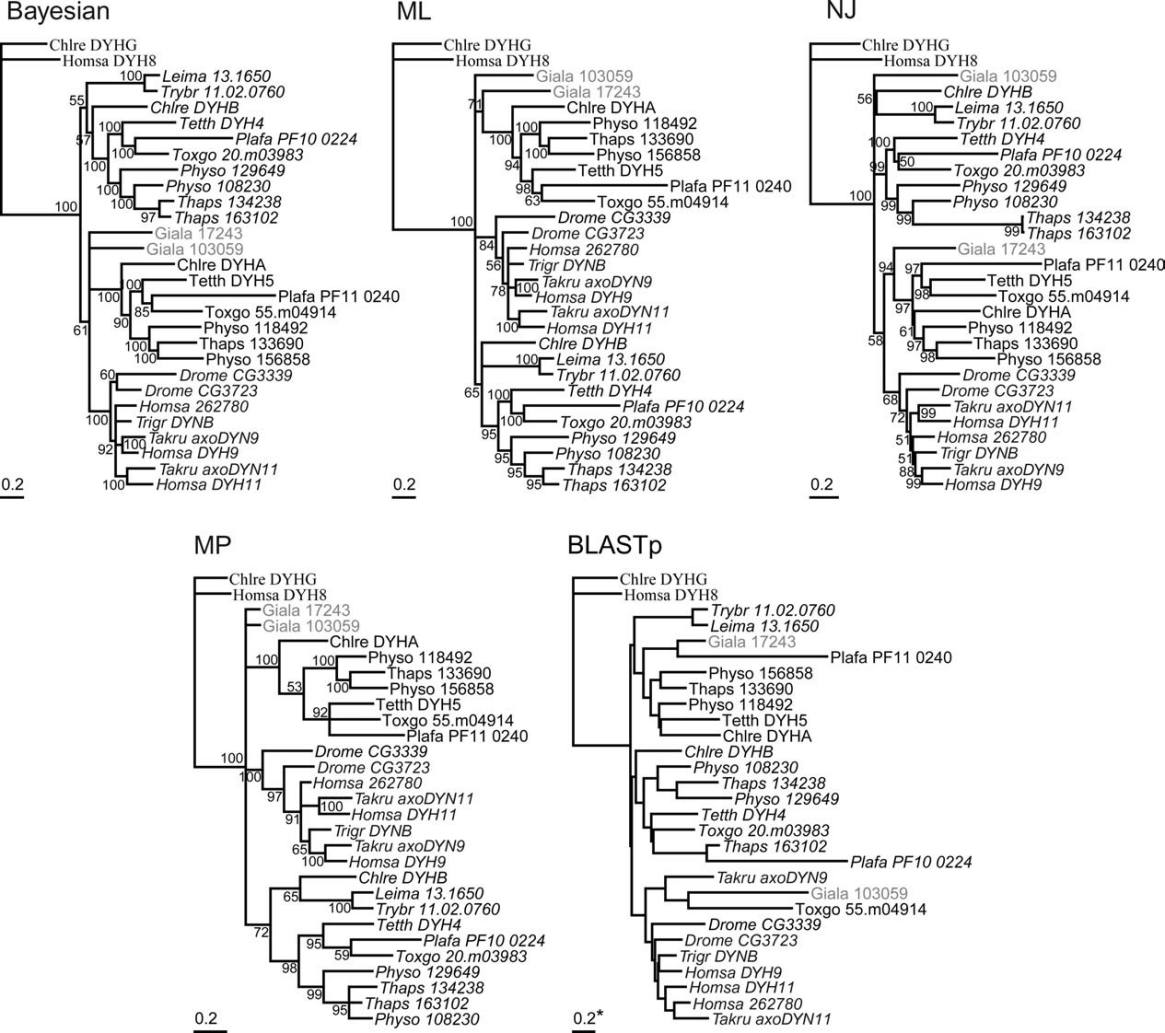
Relationships between OADβ family sequences (Figure 2) inferred by Bayesian, ML, NJ, MP or BLASTp means. Prefixes as in legend to Figure 2, with the addition of Trigr: *Tripneustes gratilla*. Trees are rooted using Chlre_DYHG and Homsa_DYH8 (OADα family members). Different fonts have been used to represent particular groups of sequences. Bars, substitutions per site; *except for BLASTp tree (see *Materials and Methods* for distance metric definition).

To test the finding of only two well-supported OAD families, we created a sub-phylogeny from a new alignment of the sequences predicted to be in the OADβ family with the addition of the OADβ from sea urchin, which was the first of the family to be sequenced (58, 59) and is therefore a good point of reference for the other members. By excluding other less-related DHC families, it is possible to increase the number of well-aligned sites for phylogenetic reconstruction of this part of the tree. The results from five different tree-building methods are shown in Figure 5. From the phylogenies, it can be clearly seen that, while there is a support for three subfamilies – metazoan OADβ, non-metazoan OADβ, and non-metazoan OADγ (*Chlamydomonas* OADα) – there is no good evidence that the metazoan and non-metazoan OADβ subfamilies are orthologous. Indeed, the Bayesian and NJ methods weakly support metazoan OADβ being an orthologue of non-metazoan OADγ. Only a tree constructed using a distance metric derived from BLASTp scores (see *Materials and Methods*) groups metazoan and non-metazoan OADβ subfamilies, which may go some way to explain the initial annotation. The tree based on BLAST scores is fundamentally different from the four others shown in Figure 5, in that, it is not based on a multiple sequence alignment and not underpinned by a model of sequence evolution. The lack of dependence on an optimized sequence alignment might be considered an advantage under certain conditions, but such methods are not generally good at reconstructing good phylogenies, and the inferred tree has several sequences in evolutionarily improbable positions. It is not clear from the phylogenies presented here whether the metazoan ancestor lost a previously present OADγ sequence, or if the metazoan OADβ subfamily represents the ancestral state, and the occurrence of the non-metazoan OADβ and OADγ subfamilies is the result of an ancient gene duplication in the ‘bikont’ lineage alone.

There is a difference between the dynein outer-arm composition of *Chlamydomonas*, *Tetrahymena* and *Paramecium*, which are 3 headed (60–63), and those of the sperm of several animal species, which contain only 2-headed OADs (64–70). The occurrence of the subfamilies in the OADβ family appears to account for this difference – species with 3-headed OADs possess OADα and members of two subfamilies of OADβ, while species with 2-headed OADs possess OADα and only one OADβ subfamily. We suggest further that the sequence data can predict the possession of 3-headed/2-headed OADs in other organisms. Hence, we expect that animal axonemes in general (not just sperm tails) will be 2 headed, whereas the axonemes of apicomplexans, diatoms and oomycetes will be 3 headed like those of *Tetrahymena* and *Chlamydomonas*. Trypanosomes and possibly *Giardia*, despite being non-metazoan organisms, possess members of only one OADβ subfamily – either through retention of the ancestral state or through secondary loss of the outermost OADγ (Figure 5) – so are predicted to have a 2-headed OAD composition reminiscent of animal axonemes. It will be interesting to see if the prediction of this simple distinction is borne out by experiment because for most axonemes the OAD composition is still unknown.

All the organisms in our analysis that encode OAD family members (which are, unsurprisingly, all the organisms that possess motile flagella/cilia) also encode identifiable orthologues of IC70 (IC2/ODA6), IC78 (IC1/ODA9) and LC1 (Figures 3 and 4B). However, no other dynein IC or LC is found exclusively and consistently in this set of organisms – in agreement with the finding of LC8 (DYNLL1/2), Tctex2 (DYNLT2) and Roadblock (DYNLRB1/2/LC7) LCs in other dynein complexes (45–47, 71–74).

The *Chlamydomonas* 2-headed IAD complex (also called I1 or *f*-dynein) contains one HC from each of the IAD-1α and IAD-1β families as well as the ICs IC138 and IC140 (36–38, 46, 75, 76). The LCs associated with the 2-headed IAD complex are generally not specific to this dynein class (LC8, Roadblock, Tctex1 and Tctex2 family members). The IAD-1α and IAD-1β families do not appear to form a single clade in our phylogeny or in previous work (29, 30). As would be expected, in most organisms that build flagella/cilia, we find homologues of IAD-1α, IAD-1β, IC138 and IC140. Interestingly, in *P. falciparum*, we could only identify a member of the IAD-1β family (not IAD-1α), and we could not detect IC138 or IC140 orthologues. However, there are highly divergent DHCs predicted in the *P. falciparum* genome that were excluded from the present analysis; so, the result is ambiguous at best.

A most surprising result of our analysis was the finding of 2-headed IAD families in the alga *O. lucimarinus*. This organism is not believed to build an axoneme in any of its life cycle stages, and it does not possess cytoplasmic dynein 1 or 2 or any of the axonemal OAD or single-headed dynein families. However, *O. lucimarinus* encodes (apparently canonical) orthologues of both IAD-1α and IAD-1β and also an IC138 homologue (a component of the same IAD I1 complex). We also found the same three proteins in the predicted proteome of the related alga *O. tauri* (data not shown), for which complete genome sequence is available (77). We can only speculate as to what function this dynein complex might be performing in these organisms. Presumably, at some point in its evolutionary history, the flagellate ancestor of *Osteococcus* must have attached a second function to the IAD-1α/IAD-1β dynein complex such that, when the flagellum was lost, the IAD genes remained under selective pressure.

The final group of axonemal dyneins is that of the ‘single-headed’ IADs. Of the three axonemal dynein groups, least is known about the members of this group (it is not even clear if all the members are indeed single headed); yet, it is the largest. The recent structural work of Nicastro et al. (70) on *Chlamydomonas* flagella found five single-headed dyneins in the 96-nm repeat of the inner arms (one of which is closely associated with the ‘2-headed’ IAD-1α/1β complex). Previous analyses have suggested six single-headed IADs (78, 79), but the molecular identities of most of the DHCs involved are unknown. Moreover, the genome of *C. reinhardtii* is predicted to encode at least nine members of this group (Figure 1). Our analysis suggests that this group is monophyletic and consists of three DHC families – although, they are less well supported than the OAD or IAD-1α/1β families. Here, we have named these families IAD-3, IAD-4 and IAD-5 to follow on from the 2-headed IAD-1α/1β families and to reflect some overlap with the weaker-supported ‘group 3’, ‘group 4’ and ‘group 5’ clades, recently identified in an analysis of sea urchin motors (30). These names do not imply a relationship to the early I2 and I3 nomenclature for IADs (80), for which there is no available sequence data. Generally, there are fewer experimental data available on the functions of the proteins of the single-headed dyneins than that for OADs or 2-headed IADs. Knockout of either IAD-4 (Tetth_DYH9) or IAD-3 (Tetth_DYH8 or Teth_DYH12) family members in *Tetrahymena* caused a reduction in cell motility (81), whereas in *Chlamydomonas*, DHC9 (IAD-3 family) enhances swimming under conditions of higher viscosity (82).

This classification of the single-headed IADs provides a framework for their study, but as yet, it provides little else by way of predictive biology. There are no ICs in our analysis that correlate with the possession of particular single-headed IAD families (or indeed IADs as a whole; Figure 3B). The dynein LC p28 is a component of the single-headed IADs and is apparently specific. However, although all organisms in our study that possess IAD HCs also possess p28, there is also an apparent homologue of the protein encoded by *C. elegans*– an organism that builds only immotile axonemes. Homologues of the recently identified subunit p38 are only found in organisms that possess the IAD-4 family, which encompasses the HC (Chlre_DHC2) with which p38 has been shown to interact in *Chlamydomonas*(83). This is suggestive of this protein being one of only two LC/LIC families that are specific to a particular dynein complex (the other being LC1 from the OAD complex as previously mentioned).

One organism in our analysis – the diatom *T. pseudonana*– lacks putative members of all five IAD families (and also IC138 and IC140). Instead, the genome of this organism encodes a canonical DYNC1H1, OADα and the two non-metazoan subfamilies of OADβ (orthologues of *Tetrahymena* OADβ and OADγ; Figure 2). The *Thalassiosira* DHC repertoire can be viewed as an ‘evolutionary experiment’, demonstrating that motile eukaryotic flagella can be formed using OADs alone. In the light of the position, diatoms are thought to occupy in the eukaryotic tree of life (Figure 3A); it is highly unlikely that the OADs of diatoms represent any kind of simplified ancestral state. Rather, it appears that *Thalassiosira* have secondarily pared down their DHC repertoire such that the axoneme – which is built only during gametogenesis – is constructed without the aid of DYNC2 and beats without the action of IADs. Interestingly, ultrastructural analysis of the gametes formed by the fern *Marsilea vestita* suggests that the reverse ‘evolutionary experiment’ has also been performed – detergent-extracted axonemes from *M. vestita* spermatozoids show no evidence of dynein outer arms (sequence data for the DHC repertoire encoded by *Marsilea* are not yet available). Similarly, mutants of *Chamydomonas* that are unable to assemble dynein outer arms are still able to build motile axonemes (84, 85). Thus, despite the high general conservation of the axonemal dyneins in organisms that build motile axonemes, particular lineages are apparently able to lose either all OADs or all IADs and still construct simplified, beating flagella.

## Materials and Methods

### DHC phylogeny

Sources and versions of the predicted protein datasets from the 24 genome sequencing projects used are given in File S1. From these datasets, we used HMMERv2.3.2 (http://hmmer.wustl.edu/) to extract all predicted proteins with a good match to the Pfam ‘dynein heavy’ (PF03028.5) domain (expectation value <10^−10^). This created a seed dataset of 151 protein sequences, which were aligned and used to create an initial phylogeny. From this phylogeny, nine DHC families were defined and used to create family-specific alignments and new HMMs. These new HMMs were used to reinterrogate the 24 predicted protein sets, and those sequences with a score >1600 to any of the nine HMMs was extracted to give a set of 170 sequences.

It is not necessary to have full sequence information to infer relationships between sequences. However, highly truncated sequences cause problems with both alignment and phylogenetic reconstruction. For this reason, sequences <1000 aa in length were removed as fragmentary. Redundancy in the remaining sequences was reduced by excluding sequences that had >95% identity to sequences already in the analysis from the same organism. The remaining 158 protein sequences were aligned using MAFFT5.861 (86) adopting the E-INS-i strategy (87) and trimmed to well-aligned blocks (2596 characters), which cover the most conserved residues of the dynein motor domain. A list of included sequences with alternative database identifiers and descriptions is provided in File S2.

A Bayesian phylogeny was inferred from the protein alignment using Metropolis-coupled Markov chain Monte Carlo method as implemented by the program MrBayes3.1.2 (88). The Whelan and Goldman (WAG) substitution matrix was used (89) with a gamma-distributed variation in substitution rate approximated to six discrete categories. Four Markov chains were run for 1 000 000 generations from a random starting tree sampling every 500 generations and with a ‘temperature’ of 0.2. Tree likelihoods appeared to reach stationary phase at around 250 000 generations, and the last 750 000 generations were used to construct the consensus tree shown in Figure 2. Support for the inferred phylogeny was produced by four methods: Bayesian partial bootstrap replicates and full bootstrap replicates under assumptions of ML, NJ and MP. For Bayesian partial replicate analysis, 10 new data matrices were created by sampling (with replacement) 1000 characters from the full alignment. These were used as above (800 000 generations), and a consensus built from the last 400 000 generations of all 10. The ML trees (100 replicates, WAG matrix, six gamma categories, alpha parameter re-estimation for each replicate) were inferred from the 2596 character matrix using the program Phymlv2.4.4 (90). The NJ trees [100 replicates, Jones, Taylor and Thornton (JTT) matrix] were inferred using software from the Phylip3.6 package (91): SeqBoot to generate resampled datasets, ProtDist for distance matrix generation and then Neighbor to infer the trees. The MP trees (100 replicates, heuristic search with tree bisection and reconnection) used the software paup4b10 (92). Consensus trees with bootstrap support for all four methods are presented in File S3. The division of phylogenies into families is subjective, but families were chosen that were (i) well supported; (ii) composed of sequence from more than one of the eukaryotic supergroups [cf. definition of kinesin superfamily (93)]; (iii) separated from the remaining sequences by a reasonably long branch; and (iv) informed by known biology (where it exists).

The OADβ sub-phylogeny (Figure 5) was inferred from a fresh alignment of OADβ sequences with the inclusion of sea urchin OADβ (accession number P23098) and two OADα sequences as an outgroup. The resulting trimmed alignment (3727 characters) was used in Bayesian (10 partial bootstraps of 2000 characters, 100 000 generations, burn-in 20 000), ML, NJ and MP analyses as above. For trees based on BLAST similarity searches, we derived a distance metric from BLASTp scores. The distance, *d*, between sequences *i* and *j* was given by:


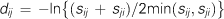


where *s_ij_* is the BLASTp score, resulting when querying sequence *i* against sequence *j*, and min(*s_ij_*, *s_ji_*) is a normalization factor for sequence length and composition given by the smaller of *s_ij_* or *s_ji_*. Note that the distance metric is symmetric (*d_ij_* = *d_ji_*) and positive (*d_ij_* = 0 when *i* = *j*; *d_ij_* ≥ 0 when *i* ≠ *j*). A matrix of distances was used to infer an NJ tree using the program Neighbor from the Phylip3.6 package (91). It is not possible to bootstrap this method.

### IC and LC phylogenies

To identify all homologues of dynein ICs and Tctex1/2 (DYNLT1/2) family LCs, we used an iterative-HMM search approach. Briefly, identified homologues from *Chlamydomonas* were used to generate seed alignments. HMMERv2.3.2 (http://hmmer.wustl.edu/) was then used to create HMMs from these alignments, which were used to interrogate a combined dataset of the 24 predicted proteomes from the chosen organisms (see File S1). Good matches to the HMM were incorporated into the alignment, and the process iterated until no further matches were identified. From the resultant datasets, redundancy was reduced by excluding sequences that had >95% identity to sequences already in the analysis from the same organism, and the remaining sequences were aligned using MAFFT5.861 (86) adopting the E-INS-i strategy (87) and trimmed to well-aligned blocks (401 and 107 characters for IC and Tctex1/2 alignments, respectively). Bayesian, ML, NJ and MP phylogenies were inferred as for DHC phylogenies (above), except that Markov chains were run for 500 000 generations (burn-in 100 000), and only four gamma categories were used. Either 10 or 50 full bootstraps of the Bayesian analyses were made for IC or Tctex1/2, respectively. Consensus trees with bootstrap support are provided in Files S4 and S5. Sequence identifiers and descriptions are provided in File S2.

### Distribution of LCs, LICs and IFT proteins

In the first instance, potential orthologues of all proteins were identified by a RBB approach using identified *C. reinhardtii* and *H. sapiens* sequences against each of the predicted protein sets (e-value cutoff in both directions: 10^−4^). Orthologues found in other organisms as RBB hits were also used to perform RBB analyses to find additional instances missed by the first analysis – any sequence *not* a RBB match to the original sequence but a RBB match to the majority of RBB matches in other organisms was considered to be a good orthologue candidate. The results of this analysis, including proteins on the OAD docking complex and radial spoke components, are presented in File S8. In the case of the gene families Tctex1/Tctex2 (DYNLT1/2; Figure 3), LC8/LC11 and LC16/LC14, additional information was gained from iterative-HMM searches and inference of phylogenies.

## Supplementary material

File S1 Sources and versions of genomic data used in this work.

File S2 Unique identifiers and descriptions for the repertoires of DHC, IC and Tctex1/2 sequences encoded in the genomes of 24 diverse eukaryotes.

File S3 Majority rule consensus trees with bootstrap support for DHCs, as inferred using Bayesian (partial sequence resampling), ML, NJ and MP approaches (see Materials and Methods).

File S4 Majority rule consensus trees with bootstrap support for dynein IC, as inferred using Bayesian, ML, NJ and MP approaches (see Materials and Methods).

File S5 Majority rule consensus trees with bootstrap support for Tctex1/2 sequences, as inferred using Bayesian, ML, NJ and MP approaches (see Materials and Methods).

File S6 Classification of the divergent Chlamydomonas dynein 206178. Tree shown is the consensus of 10 Bayesian tree inference runs with character resampling. Topology support from Bayesian, ML, NJ and MP approaches is shown next to selected nodes.

File S7 Histogram showing the distribution of scores for all sequences encoded 24 diverse eukaryotes, queried with a HMM built from an alignment of dynein IC sequences.

File S8 Reciprocal-best-BLAST hits from 24 diverse eukaryotes, queried with sequences identified in Chlamydomonas as OAD docking complex components, dynein ICs, proteins involved inIFT, dynein LCs, dynein LICs and radial spoke proteins and also components of the human cytoplasmic dynein 1 complex.
