# Supplementary material for: Dyneins Across Eukaryotes: A Comparative Genomic Analysis
Source: Traffic. 2007 Sep 26;8(12):1708–21. doi: 10.1111/j.1600-0854.2007.00646.x (PMC2239267; doi:10.1111/j.1600-0854.2007.00646.x)
Supplement: File S1 — Sources and versions of genomic data used in this work. [file tra0008-1708_8_12dfig1.pdf]

Sources and versions of genomic data used in this work.

Organism	Source[1]	Version	Web reference
Arabidopsis thaliana	TAIR	TAIR6_pep_20051108	www.arabidopsis.org/
Caenorhabdatis elegans	WormBase	WS150	www.wormbase.org/
Chlamydomonas reinhardtii	JGI	v3.0	www.chlamy.org/
Cryptosporidium parvum	cryptoDB	3.3	cryptodb.org/cryptodb/
Cyanidioschyzon merolae	C.merolae genome project	-	merolae.biol.s.u-tokyo.ac.jp/
Dictyostelium discoideum	dictyBase	Primary	dictybase.org/
Drosophila melanogaster	ENSEMBL	BDGP4.2	www.ebi.ac.uk/ensembl/
Entamoeba histolytica	geneDB	Proteins_17102005	www.genedb.org/
Giardia lamblia	GiardiaDB	-	www.mbl.edu/Giardia/
Homo sapiens	ENSEMBL	NCBI 36	www.ebi.ac.uk/ensembl/
Leishmania major	geneDB	v5	www.genedb.org/
Oryza sativa	TIGR	v4.0	www.tigr.org/tdb/e2k1/osa1/
Ostreococcus lucimarinus	JGI	v2.0	www.jgi.doe.gov/
Phaeodactylum tricornutum	JGI	v2.0	www.jgi.doe.gov/
Phytophthora sojae	JGI	v1.1	www.jgi.doe.gov/
Plasmodium falciparum	geneDB	3D7 v2.1.1	www.genedb.org/
Populus trichocarpa	JGI	v1.1	www.jgi.doe.gov/
Saccharomyces cerevisiae	ENSEMBL	SGD1	www.ebi.ac.uk/ensembl/
Schizosaccharomyces pombe	geneDB		www.genedb.org/
Takifugu rubripes	ENSEMBL	4.0	www.ebi.ac.uk/ensembl/
Tetrahymena thermophila	TGD	Predictions_Aug_2004	www.ciliate.org/
Thalassiosira pseudonana	JGI	v1	www.jgi.doe.gov/
Toxoplasma gondii	toxoDB	v4.0	www.toxodb.org/
Trypanosoma brucei	geneDB	v4	www.genedb.org/

[1] nb: Source of data does not necessarily correlate with sequencing centre(s). See individual projects for full acknowledgements of participants.
