# Supplementary figures and images for: Dyneins Across Eukaryotes: A Comparative Genomic Analysis
Source: Traffic. 2007 Sep 26;8(12):1708–21. doi: 10.1111/j.1600-0854.2007.00646.x (PMC2239267; doi:10.1111/j.1600-0854.2007.00646.x)

## Bayesian

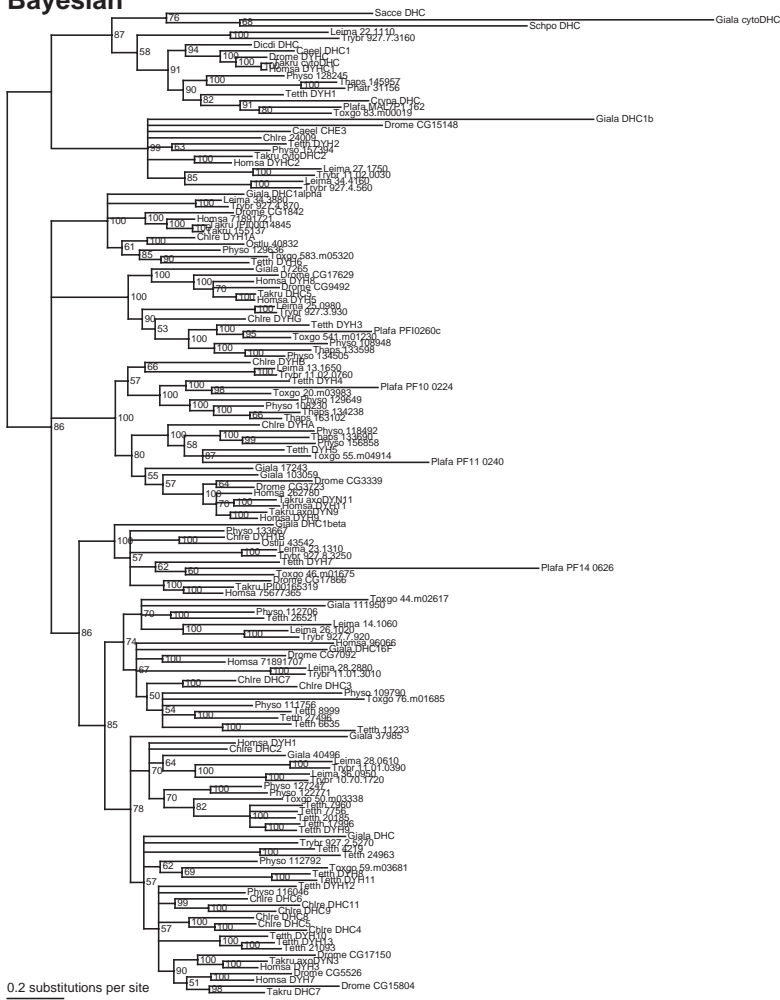

## ML

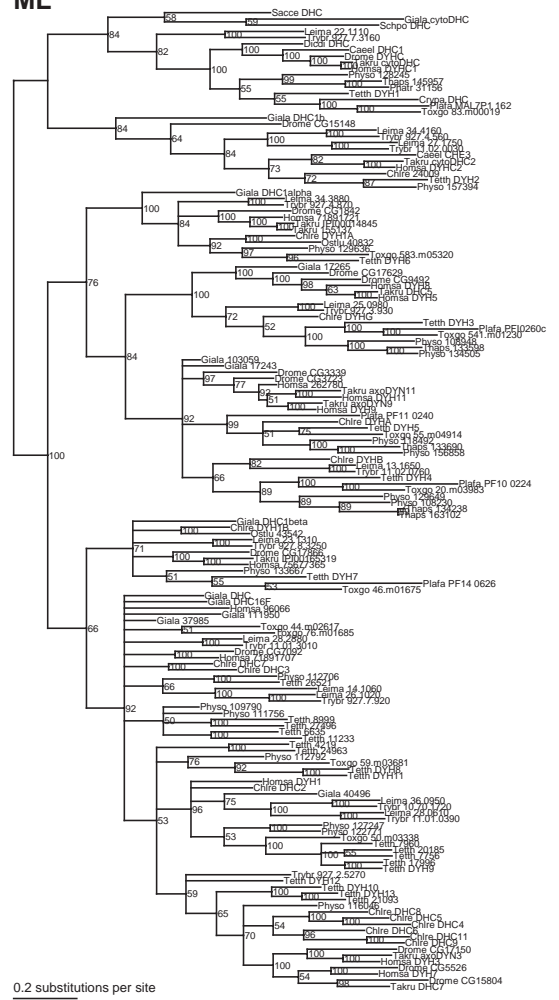

## NJ

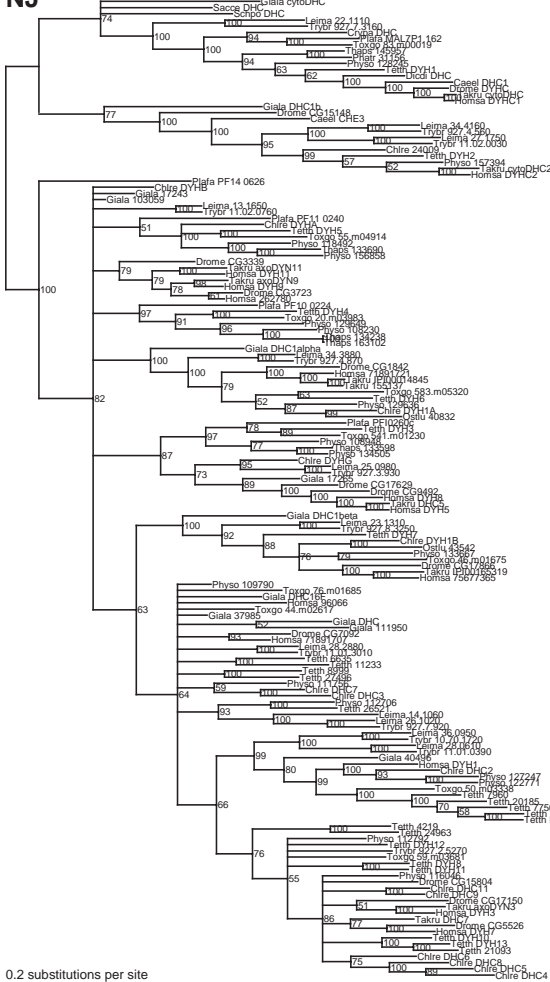

## MP

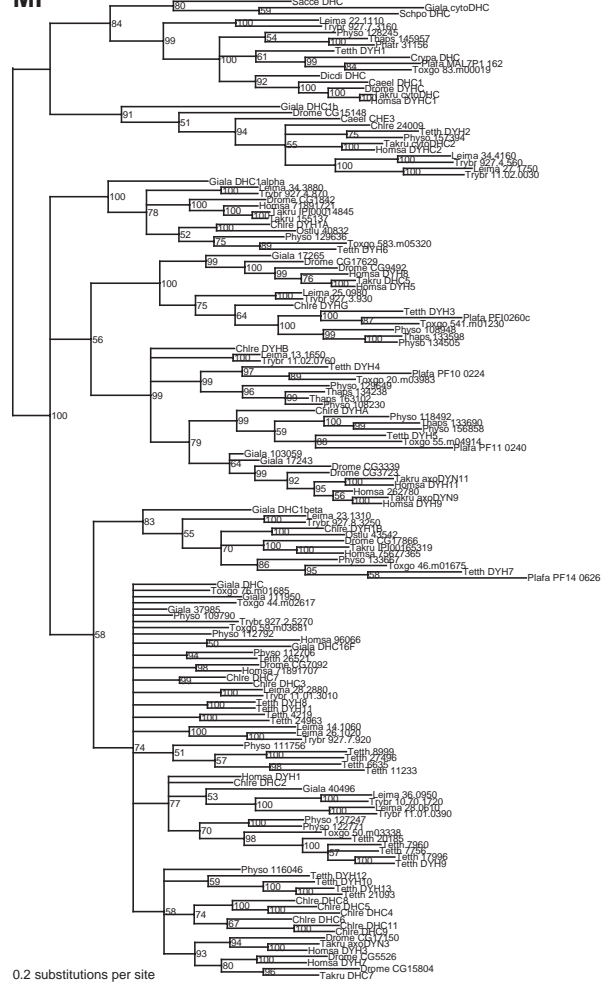

Supplement: File S3 — Majority rule consensus trees with bootstrap support for DHCs, as inferred using Bayesian (partial sequence resampling), ML, NJ and MP approaches (see Materials and Methods). [file tra0008-1708_8_12dfig3.pdf]

## Bayesian

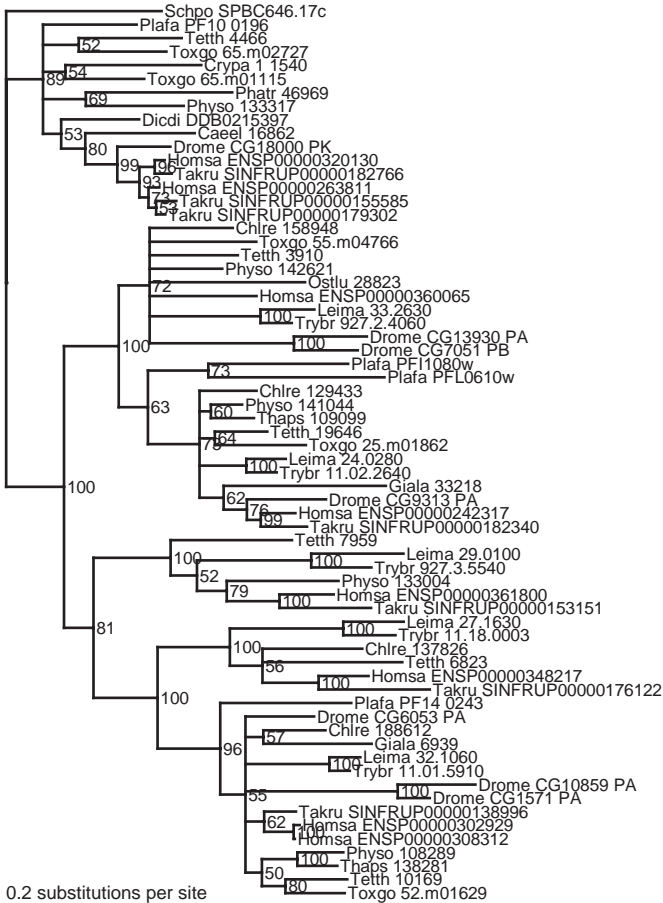

## ML

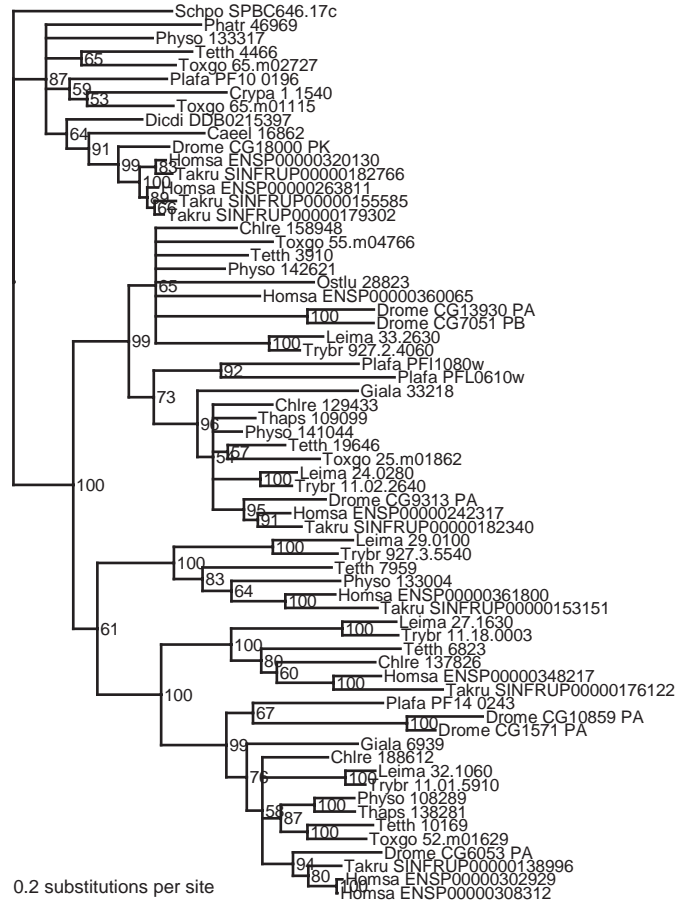

## NJ

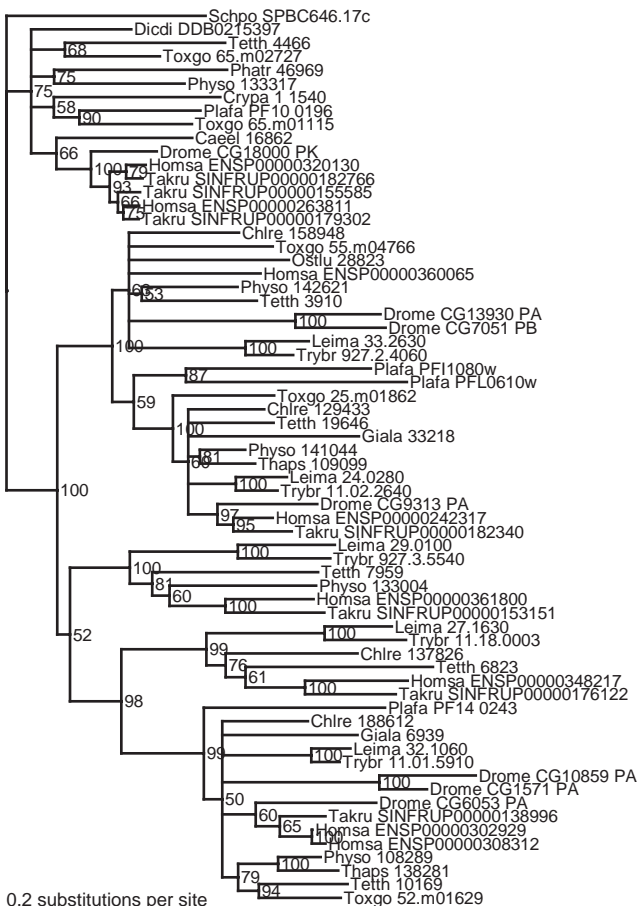

## MP

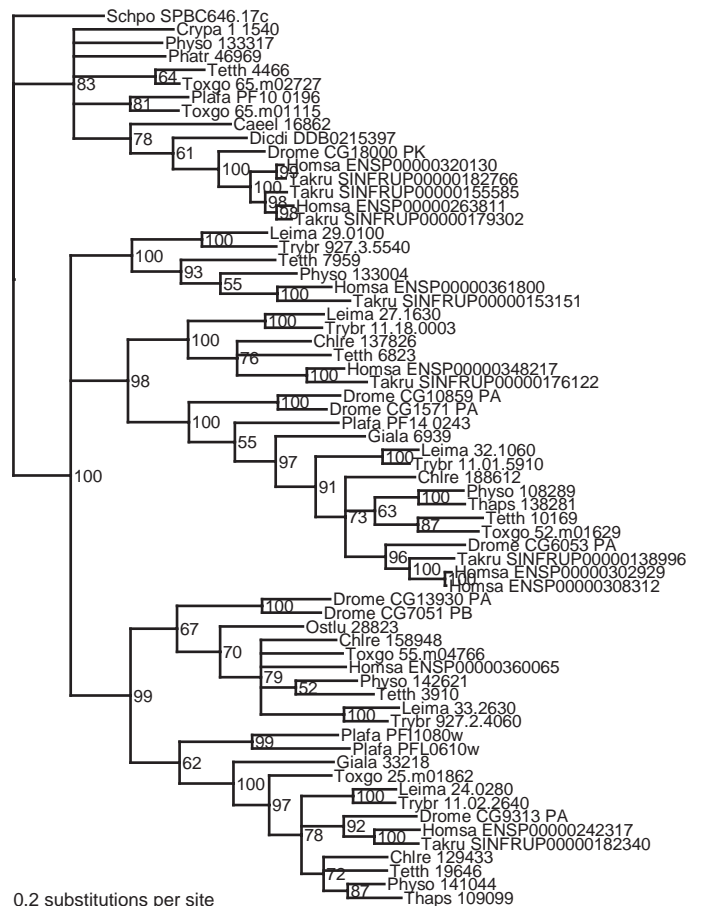

Supplement: File S4 — Majority rule consensus trees with bootstrap support for dynein IC, as inferred using Bayesian, ML, NJ and MP approaches (see Materials and Methods). [file tra0008-1708_8_12dfig4.pdf]

Bayesian

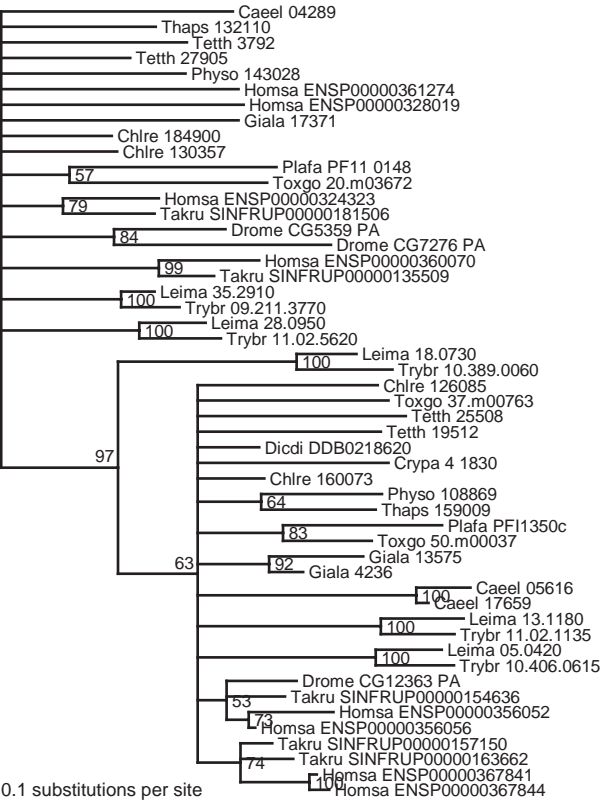

ML

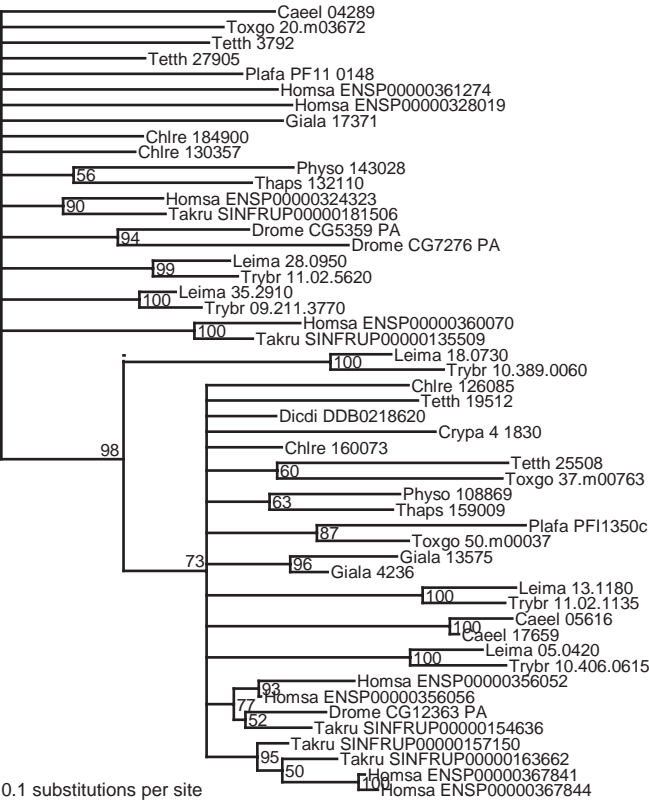

NJ

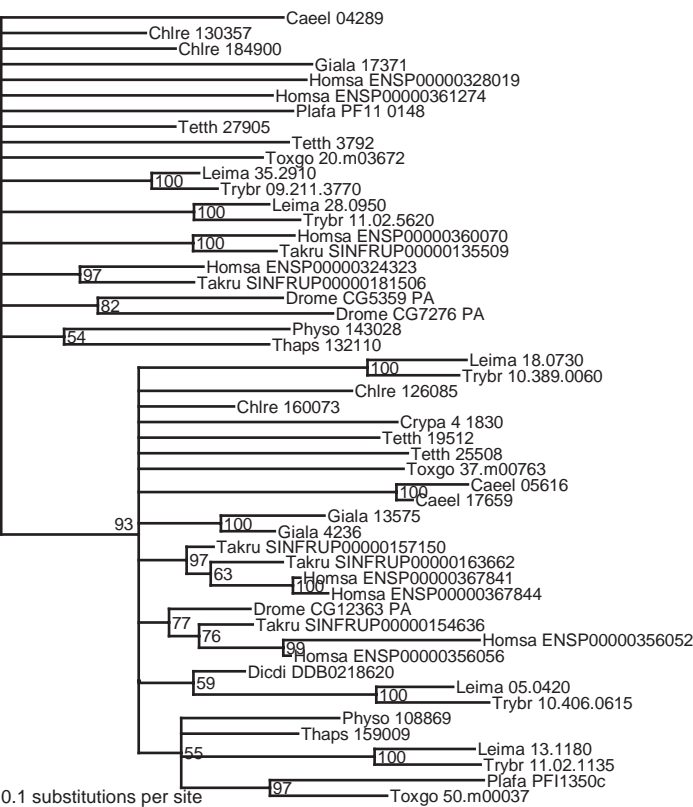

MP

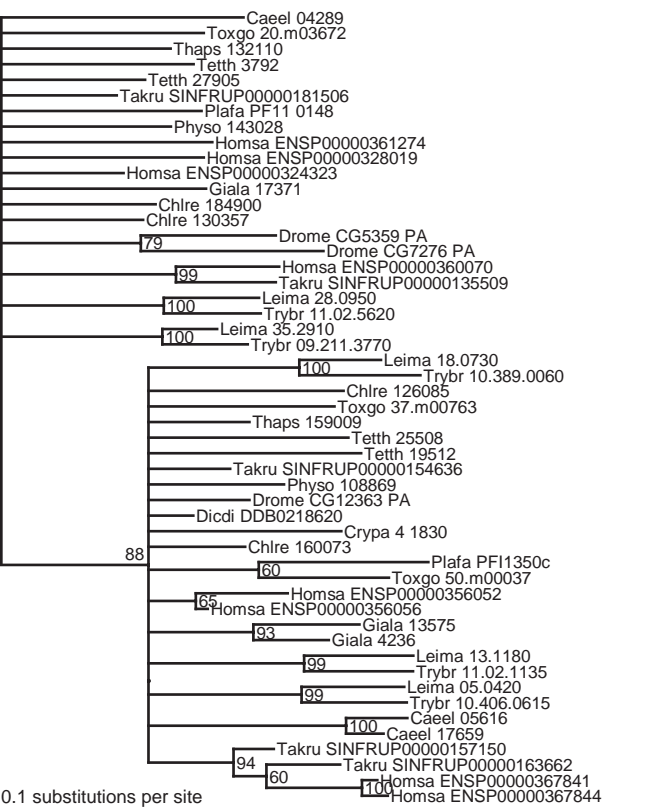

Supplement: File S5 — Majority rule consensus trees with bootstrap support for Tctex1/2 sequences, as inferred using Bayesian, ML, NJ and MP approaches (see Materials and Methods). [file tra0008-1708_8_12dfig5.pdf]

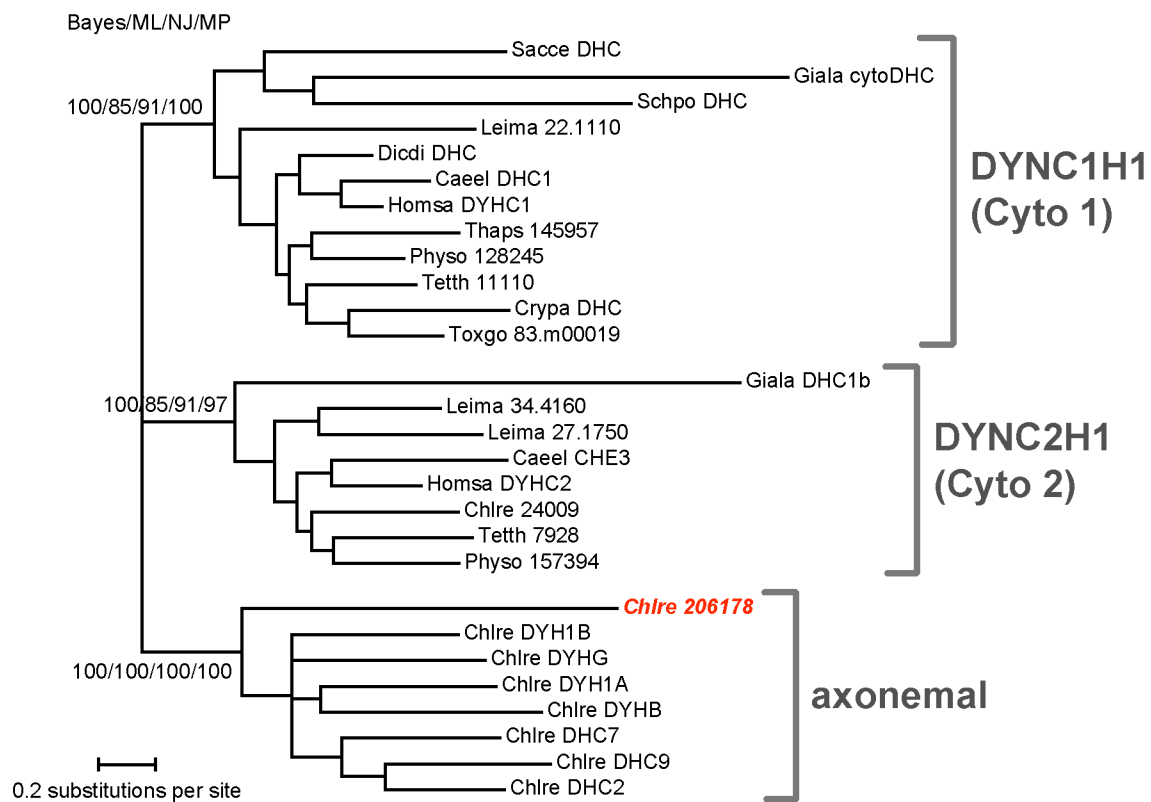

Supplement: File S6 — Classification of the divergent Chlamydomonas dynein 206178. Tree shown is the consensus of 10 Bayesian tree inference runs with character resampling. Topology support from Bayesian, ML, NJ and MP approaches is shown next to selected nodes. [file tra0008-1708_8_12dfig6.pdf]

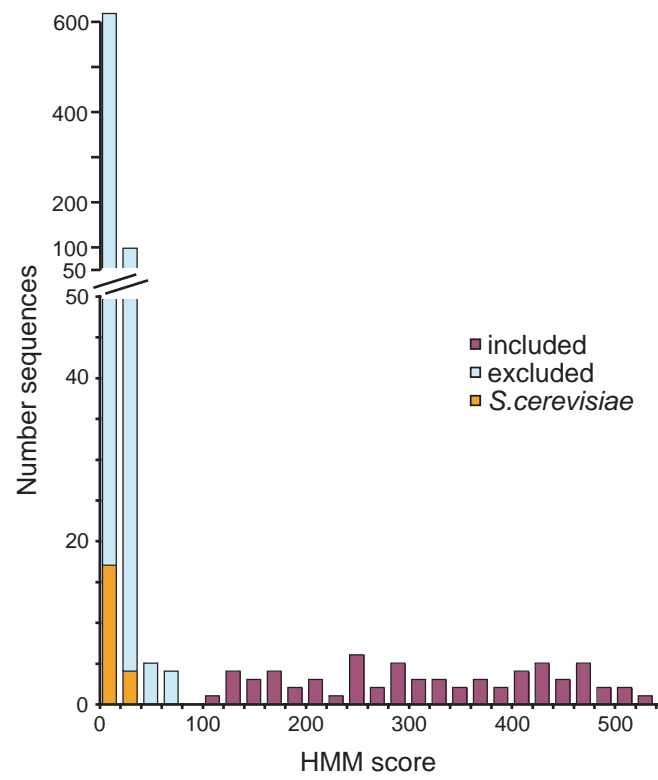

Supplement: File S7 — Histogram showing the distribution of scores for all sequences encoded 24 diverse eukaryotes, queried with a HMM built from an alignment of dynein IC sequences. [file tra0008-1708_8_12dfig7.pdf]
